# Recent Advances in the Management of Anal Cancer

**DOI:** 10.3390/healthcare11233010

**Published:** 2023-11-21

**Authors:** Laxmi Upadhyay, Michelle Hartzell, Aparna R. Parikh, Matthew R. Strickland, Samuel Klempner, Midhun Malla

**Affiliations:** 1 Department of Medicine, West Virginia University, Morgantown, WV 26506, USA; laxmi.upadhyay@hsc.wvu.edu (L.U.); michelle.hartzell@hsc.wvu.edu (M.H.); 2 Cancer Center, Massachusetts General Hospital, Boston, MA 02114, USA; aparna.parikh@mgh.harvard.edu (A.R.P.); mstrickland1@mgh.harvard.edu (M.R.S.); sklempner@mgb.org (S.K.); 3 O’Neal Comprehensive Cancer Center, The University of Alabama, Birmingham, AL 35294, USA

**Keywords:** immunotherapy, targeted therapy, personalized management

## Abstract

The incidence and mortality of squamous cell carcinoma of the anus (SCCA) is on the rise, which highlights the unmet need for advances in treatment options. The landscape of treatment for this cancer is rapidly evolving with novel combination strategies including immunotherapy, radiation therapy and biomarker-guided therapy. This review article features an overview of recent advancements in both locoregional and metastatic SCCA. The recent focus on locoregional SCCA management is to tailor treatment according to tumor burden and minimize treatment-related toxicities. Mitomycin plus either infusional 5-fluorouracil (5-FU) or capecitabine is used for first-line chemoradiotherapy (CRT), and intensity-modulated radiotherapy (IMRT) is the preferred modality for radiation for locoregional anal cancer. Locally recurrent disease is managed with surgical resection. Systemic treatment is first-line for metastatic SCCA and immunotherapy with nivolumab and pembrolizumab being included as second-line agents. Current and future clinical trials are evaluating treatments for SCCA including immunotherapy alone or in combination regimens, radiotherapies, targeted treatments and novel agents. Another critical aspect of current research in SCCA is the personalization of CRT and immunotherapies based on molecular characterization and biomarkers such as the programmed death-ligand 1 (PD-L1), epidermal growth factor receptor (EGFR) and circulating tumor DNA.

## 1. Introduction

Anal cancer is a rare gastrointestinal malignancy representing only 0.5% of new cancer diagnoses annually in the United States (US) [1]. Human papillomavirus (HPV) has been strongly linked to anal cancer, and approximately 90% of cases can be attributed to a HPV infection [2]. The incidence of anal cancer has risen at an average of 2.2% annually in the US between 2010 and 2019 and has been accompanied by a 3.9% average annual rise in the death rate from 2011 to 2020 [1]. Between 2001 and 2015, the incidence of regional disease of squamous cell carcinoma of the anus (SCCA) has nearly doubled, and the incidence of distant disease has tripled [3]. This growth in cases, coupled with the rising mortality rate, highlights the unmet need for advances in the treatment of anal cancer. This review highlights recent updates in the management of anal cancer with a focus on immunotherapy, radiation therapy, targeted treatments and circulating tumor DNA (ctDNA)-guided management.

## 2. Locoregional Anal Cancer

The completion of staging for anal cancer, after obtaining the pathologic diagnosis, guides the selection of treatment modality. The initial work-up includes an anoscopy and a digital rectal examination [4]. Staging should be completed with a computerized tomography (CT) scan of the chest, abdomen, and pelvis, or alternatively, a CT of the chest and abdomen plus magnetic resonance imaging (MRI) of the pelvis. An endoanal ultrasound of the anal canal is not currently recommended for staging in the National Comprehensive Cancer Network (NCCN) guidelines and is listed as optional in the European Society for Medical Oncology (ESMO) guidelines [4,5]. A prospective study showed a higher rate of detection of small anal cancers, of less than 2 cm, by endoanal ultrasounds compared to MRIs [6]. Endoanal ultrasounds may therefore be useful for the detection of superficial anal cancers compared to MRIs, but it should be noted that an additional CT or MRI of the pelvis is required for nodal assessment due to the lack of visualization of these regions by endoanal ultrasound [6]. The consideration of a baseline positron emission tomography (PET)/CT scan can be used to further characterize/investigate/assess regional or distal nodal involvement, tumor size and radiation planning [7,8]. Accurate staging in anal cancer is vital as disease burden is correlated with survival outcomes and guides treatment approaches. In RTOG 98-11, tumor size and lymph node involvement correlated significantly with survival outcomes [9]. Patients with bulkier T3-4N+ disease had the poorest survival rate and more frequent locoregional failure (LRF) events compared to stage T2-3N0 [9]. Lower T stage with nodal involvement had similar or better outcomes than higher T stage without nodal involvement [9]. Recent updates in the management of locoregional anal cancer focus on strategies to tailor treatment according to disease burden while minimizing treatment-related acute and long-term toxicity [10].

The first-line treatment option for locoregional anal cancer is combination chemoradiotherapy (CRT). The exception is early stage, perianal disease excluding the anal sphincter and superficially invasive SCC of the anus, for which wide local excision is a treatment option [4]. CRT in anal cancer includes treatment with mitomycin-C (MMC) and 5-fluorouracil (5-FU) based on the Nigro protocol of 1974, which revolutionized the standard of care from surgical resection to CRT alone [11]. This regimen has continued to be the current standard of care over the past 4 decades, despite several attempts to examine alternative chemotherapies in randomized control trials and particularly studies to evaluate alternatives that may improve toxicity given the common hematologic, gastrointestinal and constitutional adverse effects associated with 5-FU and mitomycin [9,12,13,14,15,16,17]. RTOG 98-11 was a phase III clinical trial that compared the regimen of 5-FU, mitomycin and radiation therapy to induction 5-FU and cisplatin followed by combination 5-FU, cisplatin and radiation therapy in predominantly non-metastatic patients (66%) [17]. The cisplatin-containing regimen failed to show benefits in progression-free survival (PFS) or disease-free survival (DFS), and a significantly higher cumulative rate of colostomy was observed (10% vs. 19%, 95% CI, 1.07–2.65; *p* = 0.02) [17]. Along with infusional 5-FU, capecitabine is a first-line chemotherapy option during CRT in nonmetastatic settings [4,18,19]. After completion of CRT, patients should be monitored for a clinical response for up to 6 months before the determination of persistent disease to ensure the full radiotherapy effect. Patients should be assessed with physical examinations and digital rectal exam every 3 months, beginning at 8–12 weeks post CRT [4]. Disease progression or persistent disease can be confirmed with tissue sampling and re-staging with CTs of the chest, abdomen and pelvis or chest and abdomen plus MRI of the pelvis [4]. Surgical management with an abdominoperineal resection (APR) is reserved as a treatment option for locally recurrent or persistent disease despite CRT [4].

In locoregional SCCA, intensity-modulated radiotherapy (IMRT) is the preferred modality for radiation therapy (RT), which utilizes variable radiation intensities that facilitate more precise mapping of the tumor target and spares nearby structures [20]. The treatment goals of IMRT are to optimize radiation duration and dose to prevent locoregional recurrence and minimize toxicity. IMRT is preferred over standard 3D-conformal radiotherapy (3D-RT) based on the results of the phase 2 RTOG 0529 trial, a multicenter prospective, randomized clinical trial that compared conventional RT to dose-painted IMRT [21]. The primary endpoint was reduction in the combined rate of grade 2+ gastrointestinal and genitourinary acute adverse events by at least 15% compared to previous results in RTOG 98-11 [17,21]. The primary endpoint was not met, but significant reductions in acute grade 2 hematologic, grade 3 dermatologic and GI toxicity were observed [17,21]. Additionally, fewer treatment breaks due to toxicity were observed, and gaps in RT have previously been associated with inferior locoregional disease control [21,22]. Despite its improved toxicity profile, retrospective studies did not show a difference in survival outcomes between patients who received conventional RT versus IMRT [23,24].

A potential strategy to also decrease treatment-related toxicity while maintaining treatment effect in patients with locoregional disease is dose reduction of chemotherapy. The JROSG 10-2 trial in Japan was a prospective phase II multicenter trial that evaluated the efficacy of reduced dose 5-FU (800 mg/m^2^/day) compared to standard doses (1000 mg/m^2^/day) in 31 patients with stage I-IIIB SCCA treated with CRT [25]. The median radiation dose was 59.4 Gy, and 97% of patients received IMRT. The primary endpoint of 2-year disease-free survival (DFS) compared to RTOG 0529 was 77.4% vs. 96% and 2-year OS was 93.5% vs. 76%. A lower rate of grade 3 or greater GI toxicity (16% vs. 21%) but a greater rate of radiation dermatitis (32% vs. 23%) was observed compared to RTOG 0529 [21,25]. Only 13% of patients required interruptions in radiotherapy, however the study results are limited by the small sample size of 31 patients and the minimum requirements for enrollment not being met. Further prospective trials are needed to evaluate the efficacy and outcomes of chemotherapy dose reduction as a toxicity-sparing strategy in CRT.

Dose escalation and radiation boost have been investigated to decrease the rate of LRF and improve survival outcomes. The phase III ACCORD trial previously investigated radiation boost after CRT with a standard dose boost of 15 Gy or high dose boost of 20–25 Gy [26]. There was a trend towards improved five-year colostomy-free survival (CFS) (77.8% vs. 73.7%, *p* = 0.067) and five-year local control (83.1% vs. 78.2%, *p* = 0.28) with high-dose radiation boost [26]. A retrospective National Cancer Database review of 10,524 patients with nonmetastatic disease between 2004 and 2015 also failed to show a benefit to OS with a higher dose of radiation of ≥54–60 Gy compared to 54 Gy in locally advanced anal cancer (HR 1.08, *p* = 0.166) [27]. A recent retrospective single-institution analysis evaluated the effect of a radiation boost of >63 Gy versus that of ≤63 Gy to the primary tumor [28]. The high-dose boost failed to show significant improvements in locoregional recurrence, 3-year OS and progression-free survival (PFS). Subgroup analyses demonstrated improvement in the 3-year CFS for T2/T3 tumors (72.6% vs. 100%, *p* = 0.008) and 3-year PFS for T1/T2 tumors (76.7% vs. 100%, *p* = 0.035). However, these improvements were at the expense of a higher rate of chronic skin toxicity (43.8% vs. 69%, *p* = 0.042). Notably, the study showed that patients who received IMRT had a significantly longer 3 years of OS compared to those who received 3D-RT (75.4% vs. 53.8%, *p* = 0.048) [28]. While radiotherapy escalation efforts have not led to meaningful improvements in clinical outcomes thus far, there may be select scenarios where this approach is beneficial. The optimal radiation dose remains an area of interest and is a focus of multiple active clinical trials (Table 1). ACT IV is a randomized 2:1 phase II clinical trial that included patients with locoregional T1-2N0 SCCA with primary tumors that are less than 4 cm in size [29]. It evaluates dose reduction of IMRT with 41.4 Gy in 23 fractions compared to standard doses with the primary outcome of 3-year LRF. This clinical trial has completed enrollment, and results are pending.

Strategies to decrease radiation-associated toxicities are an additional focus of current research on SCCA. A potential strategy to decrease hematologic toxicity is bone marrow-sparing IMRT. A prospective phase II single-arm trial utilized ^18^FDG-PET treatment with the plan of differentiating active from inactive pelvic bone marrow [30]. Radiation was delivered using VMAT (volumetric modulated arc therapy), a specialized form of IMRT that utilizes a rotational approach, and beam modulation [31]. A 19% rate of grade 3 or higher hematologic toxicities was observed, which was an improvement from 58% in RTOG 0529 [21,30]. Bone marrow-sparing radiation appears to be a promising, well-tolerated treatment with positive survival outcomes of 93% 2-year colostomy free survival (CFS) and 83.7% 2-year failure-free survival. It is being further investigated in the phase II DACG-II trial (NCT05385250).

Proton beam radiotherapy is an additional radiation therapy modality of interest hypothesized to minimize radiation-associated toxicity. It delivers radiation with a minimal exit dose, which prevents radiation exposure to nearby organs [32,33]. An open-label, prospective multicenter pilot clinical trial evaluated proton beam radiotherapy according to the dose prescription in the RTOG 0529 protocol [34]. Included patients had T1-4N0-3 disease, and the primary endpoint was feasibility of proton therapy with CRT, defined as a rate of grade 3+ dermatologic toxicity below 48%. The trial met its feasibility endpoint with a 24% rate of grade 3+ dermatologic toxicity. A prospective feasibility trial compared IMRT to proton therapy with the hypothesis that proton therapy would have a more favorable hematologic toxicity profile in the context of CRT [35]. Although patients who received proton therapy had lower pelvic bone marrow dose metrics compared to IMRT, no significant differences in hematologic toxicity were observed. The phase II DACG 5 trial is in process and further investigates proton therapy in SCCA (Table 1). If more evidence is found for the utilization of proton therapy in SCCA, it should be noted that access to a facility that offers this treatment may be a limiting factor to its implementation.

Immune checkpoint inhibitors (ICI) have been an active focus of recently published studies on patients with locoregional SCCA. A retrospective analysis of 150 SCCA patients treated with CRT noted a correlation between the strong immune marker expression of PD-1 and CD8+ tumor-infiltrating lymphocytes (TILs) with improved local control and DFS [36]. Immune checkpoint inhibitors have been shown to be more effective in patients with a higher expression of these markers at the baseline [37]. Immunotherapy is presently recommended as a second-line treatment option in the metastatic setting, but is currently being explored in locoregional setting [4]. A recent small single-institution pilot study examined the usage of neoadjuvant immunotherapy plus chemotherapy followed by concurrent radiation therapy (RT) and immunotherapy in patients with locally advanced stage II-III disease. Five patients were enrolled and received four cycles of neoadjuvant toripalimab, a monoclonal PD-1 antibody, along with docetaxel and cisplatin followed by concurrent toripalimab and RT [38]. A clinical complete response (cCR) was observed in 80% of patients, and all patients were alive at the median follow-up of 21.8 months. The observed toxicities included grade 3 dermatitis and grade 3 hematologic toxicity. ECOG-ACRIN 2165 is a prospective phase III trial that evaluates the efficacy of single agent nivolumab versus standard observation after completion of CRT in patients with regionally advanced stage IIB-IIIC disease, with a primary endpoint of DFS up to 5 years (NCT03233711). This study has completed the accrual stage, and results are pending. Table 1 summarizes the ongoing clinical trials of ICI in locoregional SCCA.

Radiation therapy may have additional value in SCCA due to its anticipated role in enhancing the effectiveness of immune checkpoint inhibitors, which are being studied in the locoregional setting. Radiotherapy acts as a sensitizer for immune checkpoint inhibitors through several mechanisms, including antigen presentation by dendritic cells [39]. Radiation therapy also increases the density of tumor-infiltrating lymphocytes, and patients with higher CD8^+^ and PD-1^+^ TIL expression who received CRT have been shown to have superior local control, DFS and OS [36,40]. All these mechanisms contribute to enhanced immunogenicity and T-cell responses that are stimulated by immune checkpoint inhibitor therapy, supporting the role of dual ICI and radiotherapy treatment. Future studies are needed to quantify the optimal dose and duration of radiation therapy treatment when paired with immune checkpoint inhibitors to achieve the most effective treatment response.

## 3. Recurrent and Metastatic Squamous Cell Anal Cancer

The incidence of metastatic SCCA is increasing. Approximately 10% of patients have metastatic disease at diagnosis, and 10–20% of patients with the localized disease treated with CRT have been shown to have metastatic recurrence [1]. Prior to 2017, data for the treatment of recurrent/metastatic anal cancer was limited to retrospective studies [41]. The landscape of metastatic SCCA management continues to evolve with recently completed prospective phase I and II clinical trials and multiple ongoing trials focused on improving survival outcomes. Carboplatin plus paclitaxel is the current preferred treatment option for the frontline management of metastatic SCCA in the NCCN Clinical Practice Guidelines (NCCN Guidelines^®^) based on the InterAACT trial (Table 2) [4]. This randomized multicenter phase II trial included 91 patients with locally advanced or metastatic SCCA in a treatment arm (carboplatin + paclitaxel, n = 45) and control arm (5-FU and cisplatin, n = 46). The median overall survival (mOS) was significantly improved in the treatment arm compared to the control (20 versus 12.3 mo, HR: 2, *p* = 0.014) [42]. Furthermore, 36% and 71% of patients in the treatment arm had severe adverse events and grade ≥3 toxicity compared to 62% and 76%, respectively, in the control arm (*p* = 0.016) [42]. This study marked a significant landmark in the management of newly diagnosed metastatic SCCA.

Docetaxel plus cisplatin and 5-FU (DCF) had been established as a potential standard treatment option based on a study by Kim et al., but this regimen had a high toxicity rate and was not well tolerated by the majority of patients involved in the study [44]. Therefore, an effort was put forth to identify whether a modified regimen with lower doses of chemotherapy would be a better option to decrease the toxicity. Two recent studies with modified DCF (mDCF) in patients with advanced SCCA were a prospective study of Epitopes-HPV01 and a phase II trial, Epitopes-HPV02. In these studies, patients received either six cycles of standard DCF (sDCF) (75 mg/m^2^ docetaxel and 75 mg/m^2^ cisplatin on day 1 and 750 mg/m^2^ per day of fluorouracil for 5 days, every 3 weeks) or eight cycles of lower doses of chemotherapy with modified DCF (40 mg/m^2^ docetaxel and 40 mg/m^2^ cisplatin on day 1 and 1200 mg/m^2^ per day of fluorouracil for 2 days every 2 weeks) depending on their age and Eastern Cooperative Oncology Group (ECOG) performance status. The final outcomes from the pooled data of 115 patients from these two studies were encouraging with an objective response rate of 87.7%, and 40.3% of patients achieving complete responses. The median PFS was 12.2 months (95% CI, 10.6–16.1) and median OS was 39.2 months (95% CI, 26.0–109.1), while no difference in OS (*p* = 0.57) or PFS (*p* = 0.99) between DCF and mDCF was observed. The 5-year PFS and OS were 24.5% and 44.4%, respectively. Patients tolerated the mDCF regimen significantly better compared to sDCF [45]. Consequently, mDCF is currently listed as one of the frontline treatment options in newly diagnosed metastatic SCCA. The regimen of 5-fluorouracil (5-FU) and a lower dose of cisplatin in patients with metastatic SCCA was studied in the FOLFCIS trial, which demonstrated an ORR of 48%, (95% CI, 32.6%–63%), median PFS of 7.1 months (95% CI, 4.4–8.6) and median OS of 2.1 months (95% CI, 16.9–28.1). Only one patient (2%) developed febrile neutropenia, and no grade 5 toxicity was reported [43]. Due to the encouraging results, this regimen is listed as an alternative first-line treatment option for recurrent or metastatic SCCA in the NCCN Guidelines^®^.

## 4. Role of Immunotherapy for Unresectable, Recurrent or Metastatic Squamous Cell Anal Cancer

Immunotherapy (ICI) currently holds a place in the management of refractory metastatic/advanced SCCA as single agents in the form of nivolumab or pembrolizumab. They are the preferred agents in the second-line space based on the NCI9673 and Keynote-158 studies. Table 3 summarizes clinical trials and descriptive studies that utilize ICI in recurrent unresectable advanced or metastatic SCCA.

### 4.1. Single Agent Immune Check Point Inhibitor (ICI)

The first phase II immunotherapy clinical trial, NCI9673, established nivolumab as a preferred regimen for patients with refractory metastatic SCCA. The objective response rate (ORR) was 24% (95% CI, 15–33%) with two complete and seven partial responses. The median PFS and OS were 4.1 months (95% CI, 3.0–7.9) and 11.5 months (95% CI, 7.1–NA), respectively. No serious adverse events were reported. This study suggested ICI might improve OS if introduced early in the treatment of metastatic SCCA [46]. The positive outcomes from this study inspired further studies in the field and opened the door for ICI in the treatment of SCCA. Pembrolizumab was studied in two clinical trials, KEYNOTE-028 (NCT02054806) and KEYNOTE-158 (NCT02628067), with promising results, promoting its use as a second-line agent for refractory SCCA [47,48]. The pooled data from both trials involving 137 patients showed an ORR of 10.9% (95% CI, 6.3–17.4%) with eight patients achieving a complete response and seven achieving a partial response. The median PFS was 2.1 months (95% CI, 2.0–2.1) and median OS was 11.7 months (95% CI, 8.8–14.5). The duration of response (DOR) exceeded 24 months in 84.6% of the patients [49].

Retifanlimab, a PD-1 inhibitor, was studied in a phase II trial (POD1UM-202) in 94 patients with advanced or metastatic SCCA who were previously treated with platinum-based therapy or were ineligible for platinum-based therapy. The ORR was 13.8% (95% CI, 7.6 to 22.5%), disease control rate (DCR) was 48.9% (95% CI, 38.5 to 59.5%), median PFS was 2.3 months (95% CI, 1.9–3.6) and median OS was 10.1 months (95% CI, 7.9-not estimable) after a median follow-up of 7.1 months [50]. Retifanlimab demonstrated an acceptable safety profile. This study further enhances the utility of ICI in the treatment of advanced or metastatic refractory SCCA. Due to promising results from the above studies, current NCCN Guidelines^®^ recommend ICI including nivolumab and pembrolizumab as second-line treatment options for metastatic SCCA, but they are not yet approved by the FDA [4].

### 4.2. Combination of ICI with Chemotherapy

The addition of ICI to chemotherapy did not significantly impact survival outcomes in the SCARCE-PRODIGE 60 study, which was a 2:1 randomized multicenter non-comparative phase II trial. This study compared the addition of atezolizumab to modified docetaxel, cisplatin, and 5-fluorouracil (mDCF) versus mDCF alone in unresectable, locally advanced, recurrent or metastatic SCCA. This chemo-immunotherapy combination failed to show a significant improvement in ORR (74.6% vs. 78%). The study also showed a 12-month PFS of 44.2% (90% CI, 33.7–54.2) versus 43.2% (90% CI, 28.5–57.0) and 12-month OS of 77.7% (95% CI, 68.1–88.7) versus 80.8% (95% CI, 68.1–95.9) in atezolizumab plus mDCF compared to mDCF alone. Furthermore, the addition of ICI was associated with a higher rate of grade 3 or higher toxicities compared to mDCF alone (59.0% vs. 36.4%) [51].

### 4.3. Novel Combination of ICI

The combination therapy of targeted EGFR and ICI has been investigated in SCCA as a novel combination. The targeted treatment of EGFR has been explored due to its high expression, which was estimated to be 85% by a study utilizing immunohistochemistry in SCCA [52]. The combination of chemotherapy with EGFR inhibitors showed promising survival outcomes in a retrospective case series and in a prospective study involving patients with metastatic SCCA [53,54]. The phase II CARACAS trial studied the combination of avelumab and cetuximab in a prospective, non-comparative randomized multicenter open-label trial in patients with unresectable, locally advanced or metastatic disease with a primary end point of ORR. After a median follow-up of 26.7 months, outcomes favored the combination regimen with an ORR of 10% (95% CI, 2.1 to 26.5) versus 17% (95% CI, 5.6 to 34.7), median PFS of 2 months (95% CI, 1.8 to 4.0) versus 3.9 months (95% CI, 2.1 to 5.6) and mOS of 13.9 months (95% CI 7.7 to 19.4) versus 7.8 months (95% CI, 6.2 to 11.2), respectively. However, the combination resulted in a higher incidence of grade 3–4 adverse events (33.3% vs. 13.3%) compared to monotherapy [55].

Another study evaluated the synergistic effect of a VEGF blockade with bevacizumab and a PD-L1 checkpoint inhibition with atezolizumab in patients with refractory metastatic SCCA. Bevacizumab was added to counter the immune evasion and suppression within the tumor microenvironment from VEGF signaling. The results of this study showed the potential benefits of combination therapy with 2 out of 20 patients (10%) having partial responses (95% CI, 1.2–32), and 11 patients (55%) having stable disease. The median PFS and OS was 4 months (95% CI, 2.6-NA) and 11.6 months (95% CI, 9.5–20), respectively [56].

The combination of anti-PD1 therapy with anti-CTLA4 therapy was evaluated in NCI9673 (Part B) in refractory metastatic SCCA. The addition of ipilimumab to nivolumab unfortunately did not significantly prolong the primary endpoint of PFS or secondary endpoint of OS. Additionally, higher treatment-related toxicities were observed with the combination regimen [57]. This highlights the importance of correlative studies to further characterize response patterns that could identify patients to be effectively targeted in clinical practice. The ongoing clinical trials using ICI in metastatic SCCA are summarized in Table 4.

## 5. Molecular Characterization and Biomarkers

With the recent advances in treating metastatic squamous cell anal cancer, immunotherapy has emerged as a novel treatment strategy, alone or in combination with chemotherapy. Further molecular characterization of tumor biology will help these newer therapies be individualized and more effective in treatment of SCCA. Phosphoinositide 3-kinases (PI3K) and AKT (AKT Serine/Threonine Kinase) are located downstream of the EGFR receptor, and their activation signals cell survival and proliferation and enhances downstream oncogenic activity [58]. HPV-positive SCCA has demonstrated frequent mutations or amplifications in the phosphatidylinositol-4,5-bisphosphate 3-kinase catalytic subunit alpha (*PIK3CA*) (30%; *p* = 0.027) or F-box and WD repeat domain containing seven (*FBXW7*) mutations (10%) compared to HPV-negative SCCA. HPV negativity was associated with frequent tumor protein P53 (TP53) (53%; *p* = 0.00001) and cyclin-dependent kinase inhibitor 2A (CDKN2A) (21%; *p* = 0.0045) mutations [59]. Mutations in *PIK3CA*, *Akt1*, *FBXW7* and phosphatase and tensin homolog (*PTEN*) loss point towards the potential for targeting the PI3K pathway [52]. A hybrid capture-based next-generation sequencing of exons from 236 cancer-related genes in SCCA demonstrated the *PI3K*/*AKT*/mammalian target of rapamycin (*MTOR*) gene amplification and homozygous deletion in 63% of cases as well as PIK3CA as the most frequent tumor alteration in SCCA reported in 40% of patients [60]. Mondaca et al. reported potentially targetable alterations in the *PI3K* pathway in 44% of SCCA tissues [43]. Another study demonstrated that PIK3CA drives anal carcinogenesis along with HPV16 oncogenes [61].

### 5.1. PD-L1 (Programmed Cell Death-Ligand-1)

PD-1 on activated T cells and PD-L1 on cancerous, parenchymal and myeloid cells mediate tumor tolerance. The activation of this axis leads to tumor-infiltrating lymphocyte (TIL) dysfunction via innate or adaptive resistance [62,63,64]. Immune checkpoint inhibitors inactivate this pathway and restore the immune system’s antitumor effects [65]. Armstrong et al. performed multiplatform testing in 311 SCCA tumor samples, and the expression of PD-1 was seen in 68.8% and PD-L1 in 40.5% of tumors [66]. Fifty-seven percent of non-metastatic SCCA showed PD-L1 expression. PD-L1 positive tumors in non-metastatic SCCA were associated with significantly better DFS and OS (*p* = 0.006 and *p* = 0.002, respectively) after definitive completion of chemoradiotherapy compared to PD-L1 negative tumors. Furthermore, the results showed an eight-fold increase in the likelihood of a complete response (CR) in patients with high PD-L1 expression levels (CPS ≥ 10%) compared to PD-L1 negative cases (OR = 8.50, 95% CI, 2.44–53.62; *p* = 0.004) [67]. In another retrospective analysis of 55 patients with SCCA, 42 patients were HPV-positive and 61.1% of patients were positive for PD-L1. There was no correlation between HPV infection and PD-L1 expression. PD-L1 positive tumors had better OS than PD-L1 negative tumors (69.3 vs. 28.3 months, *p* = 0.006), and PD-L1 expression status was an independent prognostic marker for survival (*p* = 0.012) [68]. A study by Balermpas et al. showed that patients with higher PD-1+ TIL expression had improved DFS (*p* = 0.007) and OS (*p* = 0.039) [36]. Furthermore, high HPV16INK4a and PD-L1 expressions predicted better local control (*p* = 0.011 and *p* = 0.033, respectively). This study suggests a robust explanation for the favorable clinical outcome of HPV16-positive SCCA patients harboring intense immune cell infiltration. These findings are essential to the treatment stratification with PD-1/PD-L1 immune checkpoint inhibitors to complement chemotherapy. PD-L1 expression (CPS ≥ 1) was seen in 30% of HPV-positive and 40% of HPV-negative cases [36]. PD-1 expression indicates the benefits of using ICI as a potential treatment option, but it could also be affected by HPV16 status and other molecular markers as reported by the above-mentioned studies. In the recent molecular profiling of the CARACAS trial, there was significantly better OS (HR = 0.33, 95% CI, 0.13–0.81; *p* = 0.015) and PFS (HR = 0.48, 95% CI, 0.23–1.00; *p* = 0.015) in patients with high tumor mutational burden (TMB) and PD-L1 [69]. Further studies are necessary to evaluate PD-1/PD-L1 in relation to other novel molecular markers and the potential benefits in personalized treatment.

### 5.2. Epidermal Growth Factor Receptor (EGFR)

While EGFR overexpression is extremely common in SCCA in up to 88% of cases, the EGFR mutation rates were found to be very low across multiple studies [52,66,70,71,72]. In a study with a small sample of 56 patients, most of the patients (approximately 90%) received anti-EGFR monoclonal antibodies with chemotherapy, and the response rate of any response was 41%, median PFS was 4.3 months and median OS was 16 months [54]. In another study of 17 patients where 76% of the patients received an EGFR monoclonal antibody in the second-line setting, 35% had an ORR and 24% had stable disease. The overall median PFS and OS were 7.3 months and 24.7 months, respectively [53]. The positive outcomes in these studies, as previously mentioned, suggest the need for further exploration of EGFR inhibition in metastatic SCCA. Regrettably, cetuximab did not show promising results in immunocompetent (ECOG 3205 trial) and HIV-associated SCCA (AMC045 trial) when added to standard frontline 5-FU plus cisplatin concurrent with radiation [73,74] Many studies have shown the overexpression of EGFR in SCCA, however its prognostic and predictive potential towards the personalized management of SCCA needs to be further explored in both basic science and clinical trial research.

### 5.3. Human Papillomavirus (HPV)

Human papillomavirus (HPV) is associated with SCCA in up to 91% of patients, the majority of which is HPV16 (76–94%) [75,76,77]. HPV-negative SCCA is frequently associated with TP53 mutations and is treatment-refractory [78]. Patients with HPV16INK4a tumor positivity had significantly improved survival compared to those with HPV16INK4a negativity (pooled HR:0.37, 95% CI, 0.24–0.57). Likewise, better survival was noted for HPVDNA-positive/HPV16INK4a-positive patients compared to HPV-DNA positive/HPV16INK4a negative patients (pooled HR: 0.36, 95% CI, 0.22–0.58) [76]. Another systemic review and meta-analysis also reported HPV-positive tumors had reduced locoregional recurrence (LRR) (pooled HR:0.27, 95% CI, 0.16–0.48, *p* < 0.001), improved OS (HR:0.26, 95% CI, 0.12–0.59, *p* = 0.001) and DFS (HR:0.33, 95% CI, 0.16–0.70, *p* = 0.003) compared to HPV-negative tumors after primary chemotherapy. Overall, tumors that were both HPV DNA-positive and had HPV16 positivity had the best survival outcomes [77]. All these studies demonstrate that HPV16 is the most common genotype in HPV-positive SCCA, and HPV-positive status with HPV16 is a good prognostic biomarker. These findings help in the use of combined testing of HPV and HPV16 markers for planning the individual management and follow-up of patients with SCCA.

Another implication of the HPV biomarker is its use in the screening of SCCA in high-risk populations such as men who have sex with men (MSM), human immunodeficiency virus (HIV) positive and organ transplant patients. There are no definitive guidelines regarding methods and timing of screening for anal cancers, but currently cytology is being used as a primary screening tool in high-risk populations [79,80]. There are ongoing research studies on using HPV biomarkers as a basic screening method for SCCA and high-grade squamous intraepithelial lesions (HSIL). A study by Gaisa et al. showed that high-risk HPV DNA testing significantly increases the sensitivity needed to detect high-grade dysplasia and anal cancer when used with anal cytology among high-risk populations [81]. Another systematic review and meta-analysis by Macedo et al. suggested high-risk HPV DNA can be used as a screening tool for SCCA when its usage is followed by biomarkers such as HPV16 DNA, p16 and HPV mRNA. This study showed a higher sensitivity of 92.4% (95% CI, 84.2–96.5) and a specificity of 41.7% (95% CI, 33.9–44.9) for high-risk HPV DNA followed by mRNA, but DNA HPV16 showed a higher specificity of 71.7% (95% CI, 55.3–83.8) followed by p16. However, the sensitivity of DNA HPV16 was low at 53.3% (95% CI, 35.4–70.3), while p16 had a sensitivity of 68.8% (95% CI, 47.9–84.1) [82]. Further research and evidence might be useful to establish HPV biomarkers as potential screening tools for high-grade dysplasia and anal cancer in high-risk populations.

### 5.4. Tumor-Infiltrating Lymphocytes (TILs)

Previously published studies have documented that CD8+ TILs can improve responses to chemotherapy and immunotherapy. Oropharyngeal cancer patients with HPV-positive tumors and high TILs had significantly improved OS compared to the HPV-positive cohort with low TILs [83]. The relapse-free survival rate was significantly higher with a high level of TILs compared to low or absent TILs (92% vs. 63%) in HPV-positive SCCA tumors [84]. Furthermore, a prospective study evaluating 150 patients treated with CRT demonstrated that high CD8+ and PD-1+ TILs expression predicted improved local control (*p* = 0.023 and *p* = 0.007, respectively) and DFS (*p* = 0.020 and *p* = 0.014, respectively) [36]. However, there was no improvement in OS (HR = 1.30, 95% CI, 0.72–2.36, *p* = 0.39] or PFS (HR = 1.31, 95% CI, 0.74–2.31, *p* = 0.357) in patients with high TILs in another study [69]. Data from these studies reinforces that HPV-positive SCCA has exuberant immune cell infiltration, but its overall effects on survival and personalized treatment needs to be explored further.

## 6. Other Novel Advances

Additional novel agents under investigation include vaccine therapy and targeted therapy against the TGF-β pathway. A phase II trial (NCT04287868) has investigated a novel triplet immunotherapy for HPV-related malignancies including a TGF-β blockade [85]. It included patients who progressed on a prior line of systemic chemotherapy and checkpoint therapy, if approved for their respective HPV-related malignancy. The triplet therapy included a vaccine against HPV16 E6/E7, an IL-12 immunocytokine and a bifunctional fusion protein targeting the PD-L1 and TGF-β pathways. Among the 30 patients with HPV-positive malignancies, 6 had anal cancer. The objective response rate (ORR) was 67% after the 12-month follow up period. The incidence of grade 3 adverse events was 48%, which included anemia, hematuria and GI bleeding.

Vaccine therapy targeting HPV16 is also of active interest in early phase clinical trials for the treatment of SCCA. The phase II clinical trial of the bioengineered Axalimogene filolisbac vaccine, designed to secrete Listeriolysin O and the HPV16 E7 oncoprotein, failed to meet the primary endpoint of PFS in persistent, recurrent, locoregional or metastatic disease [86,87]. A phase II trial evaluated the combination therapy of nivolumab with the experimental HPV16 peptide vaccine, ISA101, in 24 patients with HPV-positive cancer, including one with anal cancer [88]. Patients had either treatment naïve metastatic or incurable locally advanced disease. The median PFS was 2.66 months (95% CI, 2.5–9.4 months), and median OS was 15.3 months (95% CI, 10.6–27.2 months). The expression of immune and inflammatory genes correlated with positive clinical response in the study cohort. The novel SQZ-PBMC-HPV therapeutic vaccine was studied in HPV16+ cancers, including seven patients with anal cancer, in the SQZ-PBMC-HPV-101 trial [89]. Patients had a median of four prior lines of treatment. Of the total evaluable patients, 40% exhibited stable disease as the best response. This novel vaccine, which utilizes Cell Squeeze^®^ technology, has received FDA fast-track designation for HPV16+ advanced or metastatic solid tumors and is under further investigation.

## 7. Personalized Management Using Circulating Tumor DNA (ctDNA)

Circulating tumor DNA is cell-free DNA that is shed into circulation from the primary tumor and can be detected using molecular and next-generation sequencing [90]. ctDNA has tremendous relevance in the personalized management of malignancies in the form of minimal residual disease assessment, monitoring for early recurrence, molecular profiling, clonal evolution and immunotherapy response monitoring [91]. ctDNA has shown to be promising for immunotherapy response monitoring in multiple solid tumors [92,93]. ctDNA was detected 91% of the time in patients with HPV16-related advanced SCCA, and baseline HPV ctDNA levels were significantly higher in patients with metastatic disease compared to patients with locoregional recurrence (*p* < 0.001) [94]. In a study by Azzi et al., where plasma samples from 251 patients with stage I-IV metastatic SCCA were studied and analyzed, it was noted that ctDNA positivity and a higher ctDNA level were associated with metastatic disease (*p* = 0.004) [95]. The findings from the above studies suggest that the level of ctDNA correlates with tumor burden and tumor stage. In a study by Bernard-Tessier et al., chemotherapy significantly lowered ctDNA levels compared to baseline (*p* < 0.001) and 38.9% of the patients (14/36 patients) had residual HPV ctDNA despite five months of chemotherapy (95% CI, 24.8–55.1) [94]. Residual HPV16 ctDNA detected at chemotherapy completion was associated with shorter post-chemotherapy PFS (HR = 5.5, 3.4 months vs. not reached, *p* < 0.001) and a reduction of the 1-year OS rate (OR = 7.0; 95% CI, 1.5–28.5, *p* = 0.02). A low level of HPV ctDNA at the baseline correlated with longer PFS (HR = 2.1, 95% CI, 1.0–4.2, *p* = 0.04), which suggests that ctDNA response could have a role as a prognostic biomarker in anal carcinoma patients on chemotherapy [94]. A study by Cabel et al. on locally advanced SCCA demonstrated that 17% of patients displayed residual detectable HPV16 ctDNA after CRT, which was strongly associated with decreased DFS (*p* < 0.0001) [96]. The ctDNA positivity rate was associated with tumor stage (stage II and III: 64% and 100%, respectively, *p* = 0.008). Residual ctDNA levels after CRT were associated with inferior survival outcomes [96]. These results illustrate the significance of HPV16 ctDNA detection after chemotherapy and its potential value for prediction of disease recurrence and survival.

The high predictive and prognostic value of ctDNA across HPV cancers has been firmly established by different studies. The conversion from positive to negative HPV16 ctDNA by liquid biopsy was achieved in 17.9% (5/28) of patients with doublet chemotherapy in the InterAACT trial compared to 61.1% (22/36) of patients with DCF in the Epitopes-HPV02 trial [45]. Future prospective studies hold the key on whether early therapeutic intervention based on ctDNA detection ahead of radiologic recurrence would lead to improved survival outcomes. In a recent study by J. Alvarez et al., the ctDNA level was followed in stage III SCCA at the baseline, during treatment and 30 days after chemotherapy [97]. At baseline, 88% of patients had detectable ctDNA, and patients with stage III SCCA had a numerically higher baseline ctDNA compared to patients with stage I disease (26 vs. 4 mean tumor molecules per milliliter (MTM/mL), *p* = 0.08). Among 16 patients, ctDNA levels decreased significantly with treatment (19 vs. 0.9 MTM/mL, *p* = 0.05), with 50% entering molecular remission. Among 18 patients, the ctDNA level decreased (21 vs. 0.2 MTM/mL, *p* = 0.05) after treatment with 94% of patients entering molecular remission. There was no molecular recurrence or clinical recurrence with ctDNA testing at 2–4 months, 4–8 months, and 8–12 months post-CRT among the patients who had a significant decrease in ctDNA level with treatment. Another significant finding of this study was that the time taken for molecular ctDNA remission was significantly shorter than that to complete clinical response (median 30 vs. 135 days, *p* < 0.01) [97]. This signifies the potential of ctDNA as a biomarker for treatment response monitoring and prediction of early recurrence.

## 8. Future Directions

Active clinical trials in SCCA are paving the way for advancements in treatments with immunotherapy, radiation therapy dose optimization and novel combination regimens (Table 1 and Table 4). In the locoregional setting, clinical trials are evaluating neoadjuvant, concurrent and adjuvant ICI in combination with CRT. INTERACT-ION studies an induction regimen with ezabenlimab plus modified DCF [98]. CORINTH studies the addition of pembrolizumab to CRT on weeks 1, 3 or 5 of treatment [99]. RADIANCE and TIRANUS also study concurrent ICI and CRT but with an additional consolidative course of ICI thereafter [100,101]. In the metastatic setting, ICI are investigated as additions to front-line regimens, as monotherapy, in triplet regimens and in novel combinations. Pembrolizumab plus the first-line regimen of carboplatin and paclitaxel is used in EA2176 in the frontline setting for previously untreated, metastatic locally recurrent or inoperable disease [102]. POD1UM-303/InterAACT 2 is a phase III trial that builds on results of the phase II POD1UM-202 by studying the addition of Retifanlimab to carboplatin and paclitaxel [103].

Novel therapies including HPV-targeted vaccines and novel triplet immunotherapy regimens are actively being studied in early-phase clinical trials. A CD4 helper T-inducer cancer vaccine (UCPVax), previously studied in non-small cell lung cancer (NSCLC), has been investigated in combination with ICI for HPV+ cancers including SCCA in VolATIL [98]. Targeted treatment of EGFR is beingstudied in the phase I KEYNOTE-E28 trial through a bifunctional EGFR/TGFβ fusion monoclonal antibody in combination with pembrolizumab [104]. The EGFR inhibitor, cetuximab, is being studied in combination with an IL-15 receptor agonist in advanced or metastatic disease [105]. As previously discussed, ctDNA is a promising clinical tool that will be evaluated further in a planned phase II NOAC9 trial. It will compare HPV+ ctDNA-guided imaging follow-up versus standard surveillance to assess for improved detection of early treatment failure or recurrence [106]. TIRANUS explores molecular alterations as an additional outcome and includes the analysis of pre-treatment and post-treatment liquid biopsies [101]. A combined retrospective and prospective cohort study is studying HPV+ ctDNA detection in HPV-related malignancies including SCCA. It has a primary outcome of identifying the rate of detectable HPV ctDNA and a secondary outcome of accuracy of ctDNA in predicting a 24-month recurrence [107].

## 9. Conclusions

Squamous cell carcinoma of the anus (SCCA) is a rare cancer, but its incidence is rising every year. The treatment options were very limited, especially for metastatic SCCA, until a few years ago. Immunotherapy is emerging as a promising treatment for SCCA and further research is ongoing to determine its role in management of this cancer. The treatment landscape is currently evolving with the advent of studies on chemotherapy, immunotherapy, radiation therapy and novel combinations across the stages of SCCA. Along with the biomarker-driven therapeutic strategies and ctDNA-guided approaches, further progress will evolve the personalized management of SCCA.

## Figures and Tables

**Table 1:** List of ongoing and active clinical trials in non-metastatic anal cancer. Disease free survival (DFS), clinical complete response (CCR), progression-free survival (PFS), overall survival (OS), locoregional failure (LRF), disease control rate (DCR), fecal incontinence quality of life scale (FIQoL), pelvic insufficiency fractures (PIF), Gray (Gy), Fractions (F).

Clinical Trial Identifier	Focus	Name	Phase	Stage	Study Arms	Primary End Point	Status
NCT04046133	ICI	CORINTH	I	IIIA/B, T3/T4, any N, M0	Pembrolizumab + standard CRT	Safety and tolerability	Recruiting
NCT04230759	ICI	RADIANCE	II	IIB–IIIC	Durvalumab + MMC/5-FU-based CRT followed by durvalumab q4w vs. MMC/5-FU-based CRT	DFS 3 years	Recruiting
NCT05661188	ICI	TIRANUS	II	I (except anal margin), II–IIIB	Atezolizumab + tiragolumab q3w with CRT followed by 6 cycles of atezolizumab + tiragolumab	CCR and CCR rate at 26 weeks	Recruiting
NCT04719988	ICI	INTERACT-ION	II	III (TxN1 or T4N0)	mDCF + Ezabenlimab induction followed by CRT if low induction response vs. 2 cycles mDCF + Ezabenlimab followed by RT if high induction response	CCR 10 months	Recruiting
NCT05060471	ICI	N/A	II	I–III	Neoadjuvant toripalimab, docetaxel and cisplatin followed by radiotherapy and concurrent toripalimab	CCR 3 months	Recruiting
NCT05374252	ICI	N/A	III	III	Sintilimab plus CRT followed by adjuvant sintilimab for 6 months vs. standard CRT	PFS, OS CCR 6 months	Recruiting
NCT03233711	ICI	EA2165	III	IIB, IIIA–C	Post-combined modality therapy nivolumab vs. observation	DFS up to 5 years	Active, Not recruiting
ISRCTN88455282	RT	ACT3 (PLATO)	II	Locally excised anal margin T1N0	Dose-reduction; Post-operative observation only for surgical margins >1 mm vs. CRT (with 41.4 Gy in 23F) for margins ≤1 mm	LRF 3 years	Recruiting
ISRCTN88455282	RT	ACT4 (PLATO)	II	T1–2 (<4 cm) N0	Dose-reduction; 41.4 Gy 23F vs. 50.4 Gy 28F	LRF 3 years	Active, Not recruiting
ISRCTN88455282	RT	ACT5 (PLATO)	Seamless pilot II/III	T2N1-3 and T3–4, any N	Dose-escalation; 53.2 Gy, 58.8 Gy and 61.6 Gy	LRF 3 years	Recruiting
NCT04166318	RT	DECREASE	II	I or IIA	Dose-reduction; Standard dose CRT vs. De-intensified CRT (36 Gy to primary tumor with 32 Gy to elective nodal regions all in 20 fractions for T1N0 disease or 41.4 Gy to primary tumor with 34.5 Gy to elective nodal regions in T2N0)	2-year DCR ≤85%, change in FIQoL in de-instensified arm	Recruiting
NCT05385250	RT	DACG-I	II	Localized disease	Bone-marrow sparring radiotherapy	Rate of PIFs at 1 year	Recruiting
NCT05055635	RT	DACG 5 (ReRad III)	II	Recurrent disease with previous RT	Pencil beam proton therapy	Local control at 12 months	Recruiting
NCT03018418	RT	N/A	II	T2–4, any N	CRT with 5-FU, mitomycin and pencil beam proton radiotherapy	Rates of acute toxicity at 3 months	Active, not recruiting
NCT05572801	ctDNA	NOAC9	II	Locoregional	HPV+ standard of care follow-up, HPV+ ctDNA guided imaging in follow-up, HPV negative observation arm	DFS 2 years from therapy completion	Not yet recruiting
NCT04857528	ctDNA	NA	Observational	I–III, p16+	Retrospective cohort (previous blood/tumor tissue samples) and prospective cohort (blood samples, HPV genotyping, tumor tissue analysis, physical exam, PET/CT)	Rate of detectable ctHPV DNA in blood/tumor samples	Recruiting

**Table 2:** Current systemic chemotherapy frontline regimens for metastatic squamous cell anal carcinomas. CP: Carboplatin; CDDP: Cisplatin; sDCF: Standard Docetaxel + cisplatin + 5-fluorouracil; mDCF: Modified Docetaxel + cisplatin + 5-fluorouracil; RR: Response rate; mOS: Median overall survival; PR: Partial remission; CR: Complete remission; mORR: Median objective response rate; mPFS: Median progression-free survival; 5-FU: 5-florouracil; Mo: Months; W: Weeks; ECOG-PS: Eastern Cooperative Oncology Group Performance Status; ORR: Objective response rate; CI: Confidence Interval; FOLFCIS: 5-Fluorouracil (5-FU) + Leucovorin + Cisplatin; SCCA: Squamous cell carcinoma of the anus.

Study	Patients (n)	Methods	Results	Interpretation
InterAACT trial Phase II, randomized (NCT02051868)	Inoperable locally recurrent or metastatic treatment naïve (n = 91)	Cisplatin every 21 days cycle plus 5-FU, 4 times during the cycle, or to be treated with carboplatin every 28 days and paclitaxel at on days 1, 8, 15 every 28 days	CP+ Paclitaxel vs. CDDP + 5-FU RR: 59.0% vs. 57.1% mPFS: 8.1 vs. 5.7 mo (*p* = 0.375) mOS: 20 vs. 12.3 mo HR:2 (*p* = 0.014)	First-line preferred treatment option in the NCCN Guidelines^®^ for metastatic SCCA
Pooled analysis of Epitopes-HPV02 study phase II trials (NCT02402842) and Epitopes-HPV01 (NCT01845779)	Locally advanced or metastatic (n = 115)	Six cycles of DCF regimen Q3W or 8 cycles of modified DCF regimen Q2W based on age and ECOG-PS	mPFS: 12.2 mo [95% CI, 10.6–16.1] (*p* = 0.06) mOS: 39.2 mo [95% CI, 26.0–109.1] (*p* = 0.62) ORR 87.7%, CR: 40.7%	Pooled data from both studies confirms mDCF as a better tolerated and effective regimen compared to sDCF
FOLFCIS chemotherapy trial by Mondaca et al. [43]	Locally advanced, unresectable, and metastatic (n = 53)	5-FU with leucovorin plus cisplatin bi-monthly cycle	Median Follow-up: 41.6 mo RR: 48%, [95% CI, 32.6–63%] mPFS: 7.1 mo [95% CI, 4.4–8.6] mOS: 22.1 mo [95% CI, 16.9–28.1]	Safe and effective as first-line chemotherapy in advanced anal SCCA

**Table 3:** Completed clinical trials in immunotherapy for SCCA. AE: Adverse events; SAE: Severe adverse events; CTLA-4: Cytotoxic T-lymphocyte-associated protein 4; PD-1: Programmed Cell Death 1; PD-L1: Programmed Cell Death Ligand 1; EGFR: Epidermal growth factor receptor; VEGF: Vascular Endothelial Growth Factor; mFU: Median follow-up. W: Weeks, Mo: Months.

Study	Population	Agent and Setting	Targets	Outcomes	Interpretation
NCI9673, Phase II trial (NCT02314169)	Refractory metastatic previously treated with CRT (n = 37)	Nivolumab q2w (3 mg/kg)	PD-1	mFU 10.1 mo [95% CI, 9.2–12.2] ORR: 24% [95% CI, 15–33] mPFS: 4.1 mo [95% CI, 3.0–7.9] mOS: 11.5 mo [95% CI, 7.1–not estimable]	Nivolumab was well tolerated and effective monotherapy for metastatic SCCA
Pooled data from KEYNOTE-158, Phase II trial, (NCT02628067) and KEYNOTE-128, Phase 1b trial, (NCT02054806)	Metastatic and/or unresectable with prior treatment failure or intolerance to standard therapy (n = 137)	Pembrolizumab (200 mg q3w) in KEYNOTE-158 and Pembrolizumab (10 mg/kg q2w) in KEYNOTE-128 trial	PD-1	mFU: 11.7 mo ORR: 10.9% [95% CI, 6.3%–17.4%] mPFS: 2.1 mo [95% CI, 2.0–2.1] mOS: 11.7 mo [95% CI, 8.8–14.5]	Pembrolizumab is an alternative therapy for previously treated metastatic SCCA with manageable safety profile
PODIUM 202, Phase II trial, (NCT03597295)	Previously treated, advanced or metastatic (n = 94)	Retifanlimab (500 mg q4w)	PD-1	mFU: 7.1 mo [range, 0.9–19.4] ORR: 13.8% [95% CI, 7.6% to 22.5%] mPFS: 2.3 mo [95% CI, 1.9–3.6] mOS: 10.1 mo [95% CI, 7.9–not estimable]	Demonstrated clinically meaningful and durable antitumor activity
CARACAS study, Phase II trial, (NCT03944252)	Advanced with progression after one or more lines of treatment (n = 30)	Arm A: Avelumab (10 mg/kg q2w) alone or combined with cetuximab (500 mg/m^2^ q2w, Arm B)	EGFR + PD-L1	(Arm A vs. Arm B) mFU: 26.7 mo (range: 26.5–26.9) ORR: 10% [95% CI, 2.1 to 26.5] vs. 17% [95% CI, 5.6 to 34.7] mPFS: 2 mo [95% CI, 1.8 to 4.0] vs. 3.9 mo [95% CI, 2.1 to 5.6] mOS: 13.9 mo [95% CI, 7.7 to 19.4] vs. 7.8 [95% CI, 6.2 to 11.2]	Dual blockade of PD-L1 and EGFR showed a promising result in advanced SCCA
SCARCE, Phase II trial, (NCT03519295)	Unresectable with prior treatment (n = 20)	Atezolizumab+ Bevacizumab for median of 6 doses	PD-L1 + Anti-VEGF	ORR: 10% [95% CI, 1.2–32] mPFS: 4.1 mo [95% CI, 2.6-NA] mOS: 11.6 mo [95% CI, 9.5–20]	Potential benefit with combination in treatment-refractory SCCA
NCT03519295	Unresectable and metastatic (n = 97)	Arm A: atezolizumab (800 mg q2w) + mDCF q2w, Arm B: mDCF q2w	PD1 + chemotherapy	(Arm A vs. Arm B) mFU: 22.3 mo [95% CI, 20.8–24.8] ORR: 74.6% vs. 78.1%, mPFS: 44.2% 44.2% (90% CI, 33.7–54.2) vs. 43.2% (90% CI, 28.5–57.0) mOS: 77.7% (95% CI, 68.1–88.7) vs. 80.8% (95% CI, 68.1–95.9)	Addition of ICI to chemotherapy did not improve efficacy or survival outcomes
NCI9673 Part B, Phase II NCI ETCTN trial (NCT02314169)	Previously treated, unresectable or metastatic (n = 100)	Nivolumab (480 mg q4w) vs. nivolumab + ipilimumab (1 mg/kg q8w)	PD-L1 + CTLA-4	Nivolumab vs. Nivolumab + Ipilimumab; RR 17.4% vs. 21.5% (*p* = 0.89) PFS: 2.9 mo [95% CI, 1.91–3.98] vs. 3.7 mo [95% CI, 2.0–7.1] mOS: 15.4 mo [95% CI, 11.1–NA] vs. 20.0 mo [95% CI, 13.5–23.6]	Combination therapy did not demonstrate significant improvement in efficacy and survival outcomes compared to single-agent ICI

**Table 4:** List of ongoing and active clinical trials in locally advanced or metastatic anal cancer. Progression-free survival (PFS), objective response rate (ORR), treatment-emergent adverse events (TEAE), serious adverse events (SAE), maximum tolerated dose (MTD), objective response rate (ORR), Day (D), Weeks (w), Cycles (C).

Clinical Trial Identifier	Focus	Name	Phase	Stage	Study Arms	Primary End Point	Status
NCT04444921	ICI	EA2176	III	Metastatic, treatment naïve	Carboplatin and paclitaxel + nivolumab (D1 and D15 on C1 followed by D1 of each cycle) vs. standard care carboplatin and paclitaxel	PFS up to 2 years	Recruiting
NCT02919969	ICI	NA	II	IV, no limit to the number of prior therapies	Pembrolizumab q3w	Overall response rate	Active, not recruiting
NCT04472429	ICI	POD1UM-303/InterAACT 2	III	Inoperable locally recurrent or metastatic, previously untreated	Carboplatin and paclitaxel + retifanlimab vs. standard care carboplatin and paclitaxel	PFS up to 4.5 years	Recruiting
NCT04894370	ICI	SPARTANA	II	Metastatic, first-line	Radiotherapy, Spartalizumab + mDCF q2w for 8C, multimodal treatment of residual disease (including CRT), spartalizumab maintenance	PFS 1 year	Recruiting
NCT04287868	Triplet therapy: immunocytokine, bifunctional fusion protein, vaccine	NA	I/II	Locally advanced or metastatic, second-line or ineligible for first-line	Therapeutic HPV16 vaccine + NHS-IL12 tumor targeted immunocytokine + M7824 bifunctional fusion protein targeting PD-L1 and TGFβ up to 1 year	ORR	Active, not recruiting
NCT03946358	ICI, vaccine	VolATIL	II	HPV+ cancers locally advanced/metastatic, second-line	Aztezolizumab + UCPVax induction and boost phase	ORR 4 months	Recruiting
NCT04429542	EGFR, TGFβ	KEYNOTE-E28	I/Ib	Locally advanced/unresectable or metastatic, second-line, ICI-naïve	BCA101 (EGFR/TGFβ fusion monoclonal antibody) monotherapy, BCA101 + pembrolizumab	Safety, tolerability, incidence of dose limiting toxicities	Recruiting
NCT04616196	EGFR, IL-15	NA	1b/II	Locally advanced or metastatic, refractory to anti-PD-1 and platinum-based therapy	NKTR-255 (IL-15 receptor agonist) + cetuximab	Incidence TEAE and SAE, MTD, ORR	Active, not recruiting

## Data Availability

No original data was utilized for this manuscript.
